# GATA3 functions downstream of BRCA1 to promote DNA damage repair and suppress dedifferentiation in breast cancer

**DOI:** 10.1186/s12915-024-01881-6

**Published:** 2024-04-16

**Authors:** Xuejie Wang, Feng Bai, Xiong Liu, Bin Peng, Xingzhi Xu, Hongquan Zhang, Li Fu, Wei-Guo Zhu, Bin Wang, Xin-Hai Pei

**Affiliations:** 1 Guangdong Provincial Key Laboratory of Regional Immunity and Diseases, International Cancer Center, Marshall Laboratory of Biomedical Engineering, The First Affiliated Hospital, Shenzhen University Medical School, Shenzhen, 518060 China; 2 Department of Pathology, Shenzhen University Medical School, Shenzhen, 518060 China; 3 Dewitt Daughtry Family Department of Surgery, University of Miami, Miami, FL 33136 USA; 4 Guangdong Key Laboratory for Genome Stability & Disease Prevention and International Cancer Center and Marshall Laboratory of Biomedical Engineering, Shenzhen University Medical School, Shenzhen, 518060 China; 5 Program for Cancer and Cell Biology, Department of Human Anatomy, Histology and Embryology, School of Basic Medical Sciences, Peking University Health Science Center, Beijing, 100191 China; 6 Department of Pharmacology, Shenzhen University Medical School, Shenzhen, 518039 China; 7 Department of Biochemistry and Molecular Biology, International Cancer Center, Shenzhen University Medical School, Shenzhen, 518060 China; 8 Department of General Surgery, Shenzhen Children’s Hospital, Shenzhen, 518038 China; 9 Department of Anatomy and Histology, Shenzhen University Medical School, Shenzhen, 518060 China

**Keywords:** GATA3, BRCA1, DNA damage, Dedifferentiation, Breast cancer

## Abstract

**Background:**

Inadequate DNA damage repair promotes aberrant differentiation of mammary epithelial cells. Mammary luminal cell fate is mainly determined by a few transcription factors including GATA3. We previously reported that GATA3 functions downstream of BRCA1 to suppress aberrant differentiation in breast cancer. How GATA3 impacts DNA damage repair preventing aberrant cell differentiation in breast cancer remains elusive. We previously demonstrated that loss of p18, a cell cycle inhibitor, in mice induces luminal-type mammary tumors, whereas depletion of either Brca1 or Gata3 in p18 null mice leads to basal-like breast cancers (BLBCs) with activation of epithelial-mesenchymal transition (EMT). We took advantage of these mutant mice to examine the role of Gata3 as well as the interaction of Gata3 and Brca1 in DNA damage repair in mammary tumorigenesis.

**Results:**

Depletion of Gata3, like that of Brca1, promoted DNA damage accumulation in breast cancer cells in vitro and in basal-like breast cancers in vivo. Reconstitution of Gata3 improved DNA damage repair in Brca1-deficient mammary tumorigenesis. Overexpression of GATA3 promoted homologous recombination (HR)-mediated DNA damage repair and restored HR efficiency of BRCA1-deficient cells. Depletion of Gata3 sensitized tumor cells to PARP inhibitor (PARPi), and reconstitution of Gata3 enhanced resistance of Brca1-deficient tumor cells to PARP inhibitor.

**Conclusions:**

These results demonstrate that Gata3 functions downstream of BRCA1 to promote DNA damage repair and suppress dedifferentiation in mammary tumorigenesis and progression. Our findings suggest that PARP inhibitors are effective for the treatment of GATA3-deficient BLBCs.

**Supplementary Information:**

The online version contains supplementary material available at 10.1186/s12915-024-01881-6.

## Background

The integrity of the mammalian genome is under constant assault from external factors such as UV light and internal causes such as errors in DNA replication. DNA damage that is unrepaired or improperly repaired results in chromosomal rearrangement and, ultimately, cancer. DNA double-strand breaks (DSBs) are the most dangerous type of DNA damage in promoting genome instability and tumorigenesis. DNA DSBs are mainly repaired through homologous recombination (HR) and nonhomologous end joining (NHEJ) pathways. Defects in these pathways are closely associated with the development and progression of cancers, particularly hormone-related cancers including breast cancer [1, 2]. Recently, it has been reported that inadequate DNA damage repair promotes aberrant mammary epithelial cell differentiation, leading to breast cancer [3, 4]. How deficiency in DNA damage repair promotes breast cancer development and progression remains elusive.

Mammary epithelia are mainly composed of luminal and basal cells. The determination and maintenance of luminal cell fate is orchestrated by networks of transcription factors, including BRCA1 and GATA3 [5, 6]. Breast cancer comprises of, among others, two main subtypes: estrogen receptor (ER)-positive luminal and ER-negative basal-like [7]. Luminal-type tumors respond to hormone therapies. Basal-like breast cancers (BLBCs) likely originate from luminal progenitors and are poorly differentiated and aggressive [8, 9]. In addition to functioning as a transcription factor, BRCA1 is also a tumor suppressor directly involved in the repair of DNA DSBs and the maintenance of genomic stability. Functional loss of BRCA1 by germline or somatic mutation, or promoter methylation is associated with more than half of BLBCs [12–14]. BRCA1-deficient cancer cells harbor a deficiency of DNA repair by HR, which makes these cells respond well to poly ADP ribose polymerase (PARP) inhibitors. We and others have demonstrated that depletion of Brca1 in mice activates epithelial-to-mesenchymal transition (EMT) program and induces highly heterogeneous BLBCs [4, 10–12]. Interestingly, depletion of BRCA1 or its interacting proteins including FANCD2, BRG1, NUMB, and HES1 in immortalized mammary epithelial cells impairs DNA damage repair promoting aberrant luminal to basal and mesenchymal differentiation [3, 4]. However, deficiency of BRCA2, a BRCA1-interacting protein and also functions in DNA damage repair, does not cause aberrant differentiation [4]. It is poorly understood whether and how additional BRCA1-interacting proteins control DNA damage repair and differentiation in breast cancer development and progression.

GATA3, a BRCA1-interacting protein, plays a critical role in the development of multiple organs and cell lineages including the mammary gland [13–19]. In human beings, germline mutations of *GATA3* are associated with congenital hypoparathyroidism-deafness-renal disease (HDR) syndrome [20, 21], and somatic mutations of *GATA3* have been detected in ~ 15% of breast cancers [7, 22]. Expression of GATA3 is a key feature of luminal breast cancers, whereas the *GATA3 gene* is often silenced by DNA methylation [23, 24] and its expression is barely detectable in BLBCs [6, 25]. In mice, germline or epithelium-specific deletion of *Gata3* causes early lethality or severe growth defects [13–15, 26]. Loss of Gata3 in transgenic mice stimulates mammary luminal tumor progression [27, 28] and overexpression of GATA3 suppresses epithelial-mesenchymal transition (EMT) in cancer cell lines [29, 30]. We discovered that GATA3 functions downstream of BRCA1 to promote aberrant luminal to basal and mesenchymal differentiation in the induction of BLBCs [31, 32]. It has recently been reported that depletion of GATA3 may impair HR-mediated DNA damage repair by downregulating CtIP in breast cancer cells in vitro [33]. However, whether GATA3 contributes to DSB repair in mammary tumorigenesis and progression and whether GATA3 functions downstream of BRCA1 to do so remain elusive.

Depletion of either Brca1 or Gata3 in mice impairs the proliferation of mammary epithelial cells (MECs) with induction of cell cycle inhibitors preventing us from direct investigation of their functional loss in DNA damage and dedifferentiation in mammary tumorigenesis [12, 25, 31, 32, 34]. We previously demonstrated that in mice loss of p18Ink4c (p18), an INK4 family cell cycle inhibitor, promotes MEC proliferation and induces Gata3 and Brca1-proficient luminal type mammary tumors, whereas depletion of either Brca1 or Gata3 on top of p18 null background leads to the development of BLBCs with activation of EMT [12, 25, 31, 32, 35]. In the present study, we took advantage of these mutant mice to examine the role of Gata3 as well as the interaction of Gata3 and Brca1 in DNA damage repair in mammary tumorigenesis.

## Results

### Haploid loss of Gata3 or Brca1 in p18 null mice promotes DNA damage accumulation in induction of BLBCs with EMT features

**Fig. 1:**
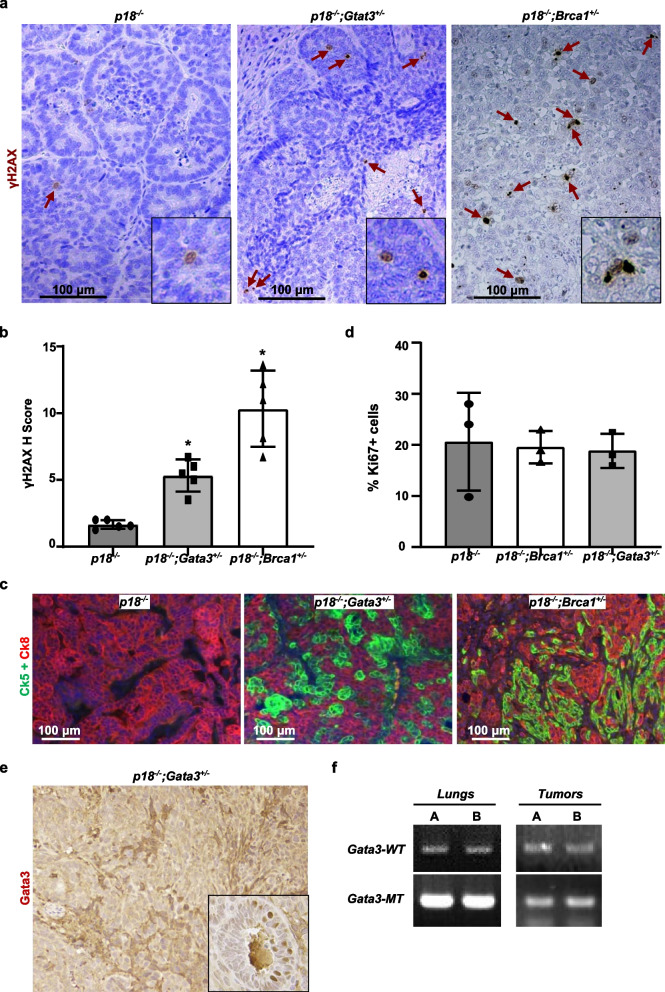
Gata3- and Brca1-deficient mammary tumors display significantly enhanced number of γH2AX-positive tumor cells, and the remaining wild-type Gata3 allele is retained in Gata3 heterozygous tumors. **a** Representative IHC analysis of mammary tumors spontaneously developed in *p18*^*−/−*^*;Gata3*^+*/−*^, *p18*^*−/−*^*;Brca1*^+*/−*^, and *p18*^*−/−*^ mice. Typical γH2AX-positive tumor cells are indicated. The inset shows the enlarged γH2AX-positive cells. **b** The *H*-scores for γH2AX in **a** were calculated. The results represent the mean ± SD of five individual tumors per group. The asterisk (*) denotes a statistical significance from *p18*^*−*/*−*^ and *p18*^*−/−*^*;Gata3*^+*/−*^ or *p18*^*−/−*^*;Brca1*^+*/−*^ samples determined by the *T*-test. **c** Representative mammary tumors were immunostained with antibodies against Ck5 and Ck8. **d** Analysis of Ki-67-positive cells in mammary tumors. Mammary tumors developed in *p18*^*−/−*^*;Gata3*^+*/−*^, *p18*^*−/−*^*;Brca1*^+*/−*^, and *p18*^*−/−*^ mice were analyzed by IHC with an antibody against Ki-67. The percentages of Ki-67-positive cells were calculated. The results represent the mean ± SD of three individual tumors per group. **e** A representative mammary tumor from *p18*^*−/−*^*;Gata3*^+*/−*^ mouse was immunostained with GATA3. Note the heterogeneous GATA3 staining in the tumor cells. The inset shows staining of a normal-like gland in the same mouse. **f** Presence of the wild-type Gata3 allele in mammary tumors of *p18*^*−/−*^*;Gata3*^+*/−*^ mice. DNA extracted from the dissected tumor samples of mice was amplified by PCR to detect wild-type (wt) and mutant (mt) alleles of Gata3. DNA from three *p18*^*−*/*−*^*;Gata3*^+/*−*^ mammary tumors were analyzed and representative results from two tumors were shown

Inspired by our finding that GATA3 functions downstream of BRCA1 to suppress aberrant luminal to basal and mesenchymal differentiation in the induction of BLBCs [31, 32], and the discovery that inadequate DNA damage repair promotes aberrant mammary epithelial cell differentiation [3], we hypothesized that GATA3, like BRCA1, also functions in DNA repair during mammary tumorigenesis. To test this hypothesis, we performed IHC analysis with the antibody against γH2AX, a marker for DNA DSBs, in mammary tumors spontaneously developed in *p18*^−/−^ and* p18*^−/−^*;Gata3*^+/−^ mice. We found that γH2AX-positive cells were barely detectable in *p18*^−/−^ tumors; however, significantly more γH2AX-positive cells were found in *p18*^−/−^*;Gata3*^+/−^ tumors (Fig. 1a, b). In line with our previous finding, we confirmed that *p18*^−/−^ mammary tumors were well-differentiated luminal type and that *p18*^−/−^*;Gata3*^+/−^ tumors were poorly differentiated basal-like with EMT features (Fig. 1c, and details in [31, 32]). These results indicate that deficiency of Gata3 promotes DNA damage accumulation and induces basal and mesenchymal differentiation in mammary tumorigenesis. As a direct comparison, we also performed a similar analysis for mammary tumors spontaneously developed in *p18*^−/−^*;Brca1*^+/−^ mice. As expected, more γH2AX-positive cells were observed in *p18*^−/−^*;Brca1*^+/−^ tumors than in* p18*^−/−^ tumors (Fig. 1a, b). Notably, the number of γH2AX-positive cells in p18^−/−^;Gata3^+/−^ tumors was less than that in *p18*^−/−^*;Brca1*^+/−^ tumors, suggesting that the efficiency of DNA damage repair by GATA3 is not as strong as that by BRCA1. We determined tumor cell proliferation in vivo and observed that the percentages of Ki-67-positive cells were comparable in *p18*^−/−^ and *p18*^−/−^*; Brca1*^+/−^ or *p18*^−/−^*; Gata3*^+/−^ tumors (Fig. 1d, Additional file 1: Fig. S1). These results are consistent with our previous discovery that loss of p18 rescues Brca1- or Gata3-deficient mammary cell proliferation [31, 35], and also suggest that GATA3 deficiency promoted γH2AX-focus formation in vivo is less likely due to cell cycle arrest. Together, these results indicate that a deficiency of Gata3, like that of Brca1, impairs DNA damage repair and promotes aberrant basal and mesenchymal differentiation in mammary tumorigenesis and progression.

We then performed IHC for *p18*^−/−^*;Gata3*^+/−^ mammary tumors and found that the expression of GATA3 was highly heterogeneous, some of the cells expressed GATA3, and some did not (Fig. 1e). We analyzed genomic DNA isolated from tumors and observed that the remaining wild-type Gata3 allele was retained in all three *p18*^−/−^*;Gata3*^+/−^ mammary tumors tested (Fig. 1f). Given that the remaining wild-type Brca1 allele was lost in CK5-positive basal-like cells, not in CK5-negative cells, in Brca1 heterozygous mammary tumors [35], and that GATA3 functions downstream of BRCA1 to suppress breast cancer [32], it will be interesting to determine if LOH of Gata3 occurs in certain groups of Gata3 heterozygous mammary tumor cells.

### Depletion of Gata3 in luminal tumor cells promotes DNA damage accumulation and produces BLBCs

**Fig. 2:**
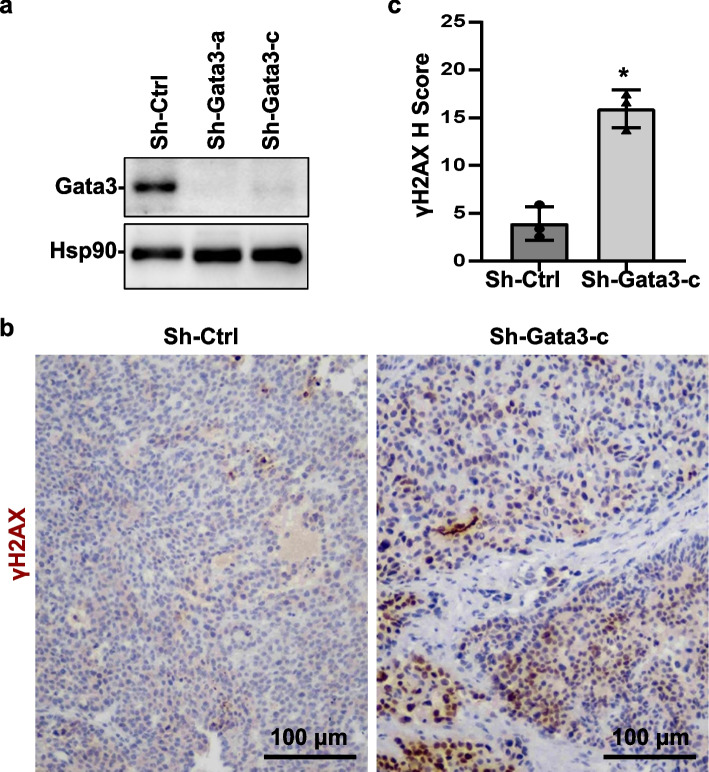
Depletion of Gata3 in MMTV-PyMT luminal tumor cells enhances DNA damage in tumorigenesis. **a** Luminal mammary tumor cells from MMTV-PyMT mice were infected with psi-LVRU6GP-control (sh-Ctrl) or psi-LVRU6GP-Gata3 targeting different sequences of mouse Gata3 (sh-Gata3-a and sh-Gata3-c), selected with puromycin, and analyzed for Gata3 expression. **b** MMTV-PyMT luminal tumor cells infected with sh-Ctrl and sh-Gata3-c were transplanted into the mammary fat pads (MFPs) of female NCG mice. Tumors formed 8 weeks after transplantation were immunostained with an antibody against γ-H2AX. **c** The *H*-scores for γ-H2AX in **b** were calculated. The results represent the mean ± SD of three individual tumors per group. The asterisk (*) denotes a statistical significance from sh-Ctrl and sh-Gata3-c samples determined by the *T*-test

Taking advantage of the luminal type mammary tumor model system we established [31, 32], we directly test if Gata3 deficiency impairs DNA damage repair in luminal tumor cells in vivo. We knocked down Gata3 in MMTV-PyMT tumor cells that were isolated and screened from MMTV-PyMT mammary tumors and were confirmed as Gata3 and Brca1 proficient (*Gata3*^+/+^*;Brca1*^+/+^) luminal type before and after transplantation into mammary fat pads (MFPs) of recipient mice (Fig. 2a, and details in [31, 32]). We transplanted MMTV-PyMT luminal tumor cells into MFPs of mice and performed IHC analysis for newly generated mammary tumors. We found that the tumors generated by Gata3-depleted cells displayed significantly more γH2AX-positive cells than tumors generated by control cells (Fig. 2b, c, Additional file 1: Fig. S2). Together with our previous finding that depletion of Gata3 in these luminal tumor cells promotes basal-like differentiation in tumors newly generated [31], these data demonstrate that depletion of Gata3 in luminal tumor cells also impairs DNA damage repair and promotes basal-like differentiation.

### *Depletion of GATA3 in breast cancer cells impairs DNA damage response *in vitro

**Fig. 3:**
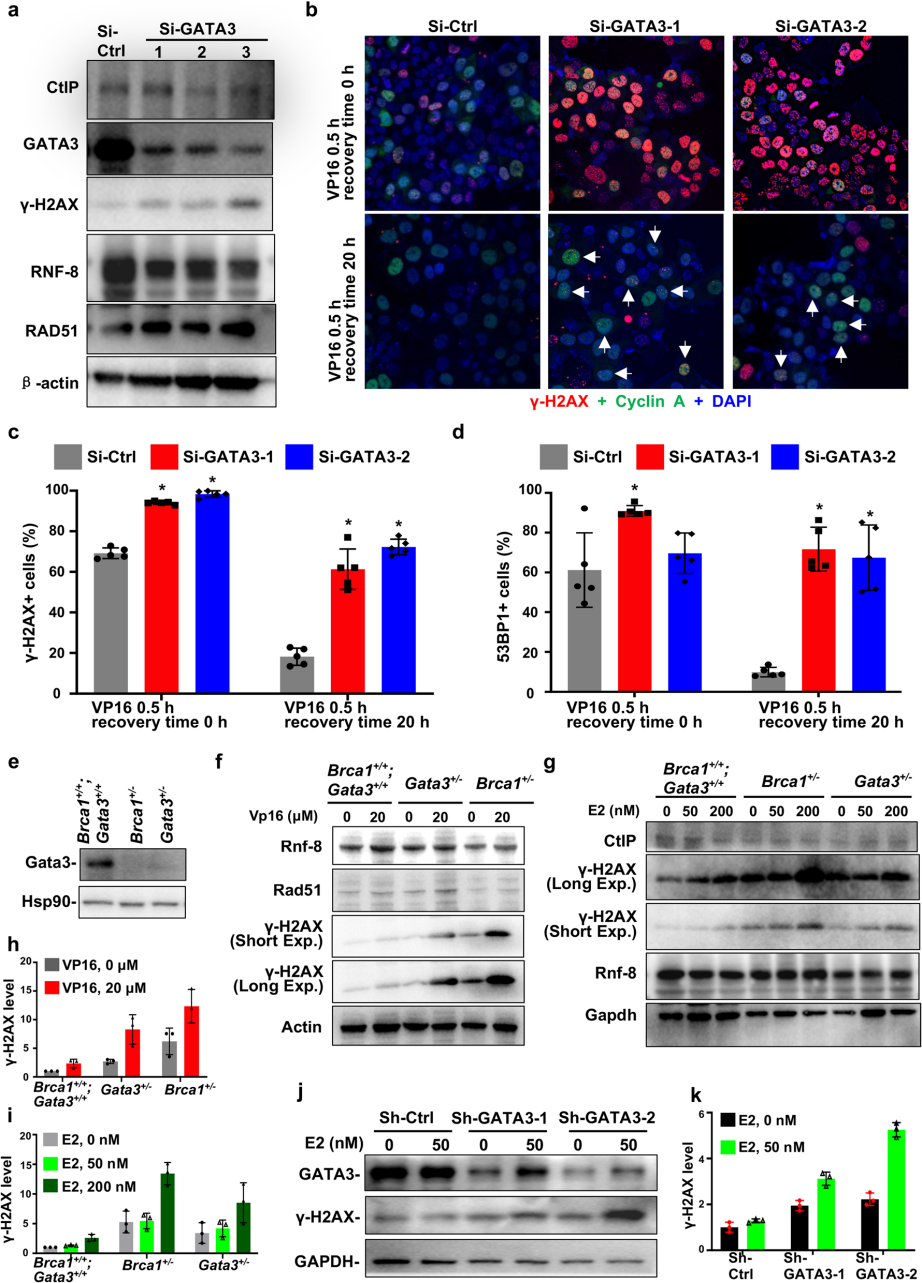
Depletion of GATA3 in breast cancer cells impairs DNA damage repair in vitro. **a** Human luminal breast cancer cell line, T47D, was transfected with Si-GATA3-1, Si-GATA3-2, Si-GATA3-3, or Si-control (Si-Ctrl) and analyzed. **b**–**d** T47D cells were transfected with Si-GATA3-1, Si-GATA3-2, or Si-Ctrl for 32 h, treated with VP16 at a concentration of 10 μM for additional 0.5 h, allowed to recover for 0 or 20 h, and then immunostained with an antibody against γH2AX and cyclin A (**b**, **c**) or 53BP1 (**d**). Representative γH2AX- and cyclin A-positive cells were shown. Note relative to T47D-Si-Ctrl cells, T47D-Si-GATA3-1 and T47D-Si-GATA3-2 cells retain high levels of γH2AX after 0- and 20-h recovery. γH2AX and cyclin A doubly positive cells are indicated by white arrows. **c** Quantification of γH2AX-positive cells in **b**. Only cells with at least 5 γH2AX foci were counted as positive cells. At least 200 cells were counted for each sample. The results represent the mean ± SD of five randomly selected fields per group. The asterisk (*) denotes a statistical significance from Si-Ctrl and Si-GATA3-1 or Si-GATA3-2 samples determined by the *T*-test. **d** Quantification of 53BP1-positive cells. Only cells with at least 3 53BP1 foci were counted as positive cells. At least 200 cells were counted for each sample. The results represent the mean ± SD of five randomly selected fields per group. **e** Analysis of Gata3 levels in MMTV-PyMT (*Gata3*^+/+^*;Brca1*^+/+^), *p18*^*−/−*^*;Gata3*^+*/−*^* (Gata3*^+*/−*^), and *p18*^*−/−*^*;Brca1*^+*/−*^* (Brca1*^+*/−*^) mouse mammary tumor cells. **f**, **g** *Gata3*^+/+^*;Brca1*^+/+^, *Gata3*^+*/−*^, and *Brca1*^+*/−*^ tumor cells were treated with VP16 (**f**) for 3 h, or E2 (**g**) for 24 h. **h**, **i** The expression of γ-H2AX in **f** was quantified in **h** and that in **g** was quantified in **i**. **j**, **k** T47D-Sh-Ctrl, T47D-Sh-GATA3-1, and T47D-Sh-GATA3-2 cells were treated with E2 for 1 h, and the expression of genes was determined (**j**) and quantified (**k**). The blots, images, and graphs represent data from at least three independent experiments

It has been reported that depletion of GATA3 in MCF7 cells reduces the expression of CtIP and impairs etoposide-induced DNA damage repair and that the overexpression of WT GATA3, but not the overexpression of a few mutants including D335E, promotes HR [33]. However, since MCF7 cells harbor a heterozygous mutant GATA3 D335E allele and express a barely detectable WT GATA3 protein [22, 32, 36], whether the loss of function of WT GATA3 impacts DNA damage repair remains to be confirmed. We knocked down GATA3 in T47D cells expressing WT GATA3 and harboring a mutant p18 [36, 37] and noticed that depletion of GATA3 downregulated the expression of CtIP and upregulated the expression of γH2AX (Fig. 3a, Additional file 1: Fig. S3). We also checked the expression of RNF-8 and Rad51, both of which play a critical role in DSB repair. We found that the knockdown of GATA3 slightly reduced the expression of RNF-8 and enhanced that of Rad51 (Fig. 3a). We then challenged these cells with etoposide (VP16), a widely used DSB-generating agent (4, 33). We noticed that 20 h after VP16 treatment the percentages of γH2AX- or 53BP1-positive cells were significantly higher in Si-GATA3 cells than in Si-control cells, indicating that depletion of WT GATA3 results in less efficient γH2AX or 53BP1 clearance and that GATA3 deficiency impairs DNA DSB repair (Fig. 3b–d, Additional file 1: Fig. S3, S4). We also determined if the treatment affects the number of T47D cells expressing cyclin A, an S-phase and cell proliferation marker. We observed that the percentages of cyclin A-positive cells were comparable in T47D-Si-GATA3 and T47D-Si-control cells treated with VP16. Notably, after VP16 treatment, some of the γH2AX-focus-positive T47D-Si-GATA3 cells co-expressed cyclin A (Fig. 3b, Additional file 1: Fig. S3b). These results also suggest that the VP16-induced higher levels of γH2AX in Gata3-deficient cells are less likely due to cell cycle arrest. In accordance with the findings derived in GATA3-deficient T47D cells, we also determined the effect of VP16 in the induction of γ-H2AX and 53BP1 in GATA3-deficient U2OS cells and obtained similar results (Additional file 1: Fig. S5).

We then analyzed primary murine mammary tumor cells isolated, screened, and characterized in our previous studies [31, 32]. Since* p18*^−/−^ luminal tumor cells proliferate very slowly in vitro and weakly generate luminal tumors when transplanted in vivo [32], we utilized a few *Gata3*^+/+^*;Brca1*^+/+^ luminal tumor cell lines isolated from MMTV-PyMT mammary tumors (Fig. 3e) (this study and reference [31]). We confirmed that *Gata3*^+/+^*;Brca1*^+/+^ mammary tumor cells expressed high levels of GATA3 and *Brca1*^+/−^ (*p18*^−/−^*;Brca1*^+/−^) or *Gata3*^+/−^ (*p18*^−/−^*;Gata3*^+/−^) mammary tumor cells expressed very weak GATA3 (Fig. 3e), as we previously described [31, 32]. We found that the treatment of* Gata3*^+/−^ (*p18*^−/−^*;Gata3*^+/−^) mouse mammary tumor cells with VP16 led to significantly more γH2AX than the treatment of *Gata3*^+/+^*;Brca1*^+/+^ (MMTV-PyMT) tumor cells (Fig. 3f, h). As a control, we conducted a similar treatment for *Brca1*^+/−^ (*p18*^−/−^*;Brca1*^+/−^) mouse mammary tumor cells and also observed a significant increase of γH2AX in *Brca1*^+/−^ cells relative to that in* Gata3*^+/+^*;Brca1*^+/+^ cells (Fig. 3f, h). Interestingly, VP16 did not clearly alter the expression of Rnf-8 and Rad51 (Fig. 3f). These results confirm the role of Gata3 loss, like Brca1 loss, in inducing DNA DSBs in tumor cells.

We then tested whether depletion of GATA3 in mammary tumor cells impairs DNA damage response to estrogen, a hormone fluctuating during the menstrual cycle and inducing DNA DSBs [38]. We found that in response to estrogen (17β-estradiol, E2) *Gata3*^+/−^ and *Brca1*^+/−^ mouse mammary tumor cells expressed higher levels of γH2AX than *Gata3*^+/+^*;Brca1*^+/+^ tumor cells (Fig. 3g, i). Consistently, the expression of CtIP in *Gata3*^+/−^ tumor cells was lower than in *Gata3*^+/+^*;Brca1*^+/+^ cells (Fig. 3g). T47D-sh-GATA3-1 and T47D-sh-GATA3-2 cells expressed more γH2AX than T47D-sh-control cells when they were treated with E2 (Fig. 3j, k). These data demonstrate that the depletion of GATA3 or BRCA1 promotes DSB-repairing defects induced by estrogen.

### Depletion of Gata3 or Brca1 promotes estrogen-induced DNA damage in mammary tumorigenesis

**Fig. 4:**
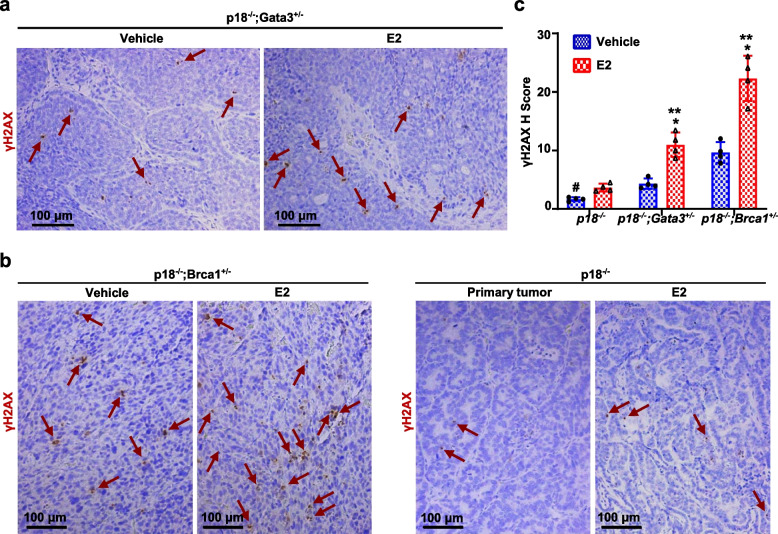
Depletion of Gata3 or Brca1 promotes estrogen-induced DNA damage in mammary tumorigenesis. **a**, **b** Representative IHC analysis of regenerated *p18*^*−/−*^*;Gata3*^+*/−*^, *p18*^*−/−*^*;Brca1*^+*/−*^, and *p18*^*−/−*^ mammary tumors treated with or without E2. Typical γH2AX-positive tumor cells are indicated. **c** The *H*-scores for γH2AX in **a** and **b** were calculated. The results represent the mean ± SD of four individual tumors per group. The asterisk (*) denotes a statistical significance from E2 and placebo-treated samples determined by the *T*-test. The asterisk (**) denotes a statistical significance from E2-treated *p18*^*−*/*−*^ and *p18*^*−/−*^*;Gata3*^+*/−*^ or *p18*^*−/−*^*;Brca1*^+*/−*^ samples determined by the *T*-test. Note since *p18*^*−*/*−*^ tumor cells did not generate tumors when transplanted without exogenous E2, we calculated γH2AX *H* scores from primary tumors as an alternative control for placebo-treated *p18*^*−*/^^*−*^ tumors (indicated by #)

Since estrogen is an intrinsic source of the induction of DNA damage in mammary epithelial and tumor cells under physiological condition, we then transplanted *p18*^−/−^ (*Gata3*^+/+^*;Brca1*^+/+^), *p18*^−/−^*;Gata3*^+/−^ (Gata3-deficient), and *p18*^−/−^*;Brca1*^+/−^ (Brca1-deficient) mammary tumor cells into MFPs of the recipient mice who were simultaneously administered estrogen or vehicle. We found that the number of γH2AX-positive cells was significantly more in estrogen-treated *p18*^−/−^*;Gata3*^+/−^ and *p18*^−/−^*;Brca1*^+/−^ tumors than in vehicle-treated counterparts (Fig. 4a–c). Because* p18*^−/−^ luminal tumor cells did not generate tumors when they were transplanted into MFPs without exogenous estrogen [32], we were unable to determine and compare the effect of estrogen in inducing DNA damage in *p18*^−/−^ (*Gata3*^+/+^;*Brca1*^+/+^) luminal type tumorigenesis. Nevertheless, the number of γH2AX-positive cells in estrogen-treated *p18*^−/−^ tumors was significantly less than the number of γH2AX-positive cells in estrogen-treated* p18*^−/−^*;Gata3*^+/−^ and *p18*^−/−^*;Brca1*^+/−^ tumors (Fig. 4a–c), indicating depletion of Gata3 or Brca1 promotes estrogen-induced DNA damage in tumorigenesis. The finding that Brca1 depletion promotes estrogen-induced DNA damage in vivo is consistent with the previous observation that BRCA1 deficiency exacerbates estrogen-induced DNA damage in vitro [38]. Interestingly, estrogen-treated *p18*^−/−^*;Gata3*^+/−^ tumors exhibited less γH2AX-positive cells than estrogen-treated *p18*^−/−^*;Brca1*^+/−^ tumors. This result is in line with the findings derived from primary *p18*^−/−^*;Gata3*^+/−^ and *p18*^−/−^*;Brca1*^+/−^ mammary tumors (Fig. 1), suggesting Gata3-mediated DNA damage repair may not be as efficient as Brca1-mediated repair in tumor development. Taking into consideration our previous discovery that estrogen promotes activation of EMT in Gata3- or Brca1-deficient tumors accelerating mammary tumorigenesis and metastasis [32, 39], these data demonstrate that deficiency of Gata3, like that of Brca1, promotes estrogen-induced DNA damage and estrogen-activated aberrant differentiation in mammary tumorigenesis and progression.

### Reconstitution of Gata3 improves DNA damage repair in Brca1-deficient mammary tumor cells and tumorigenesis

**Fig. 5:**
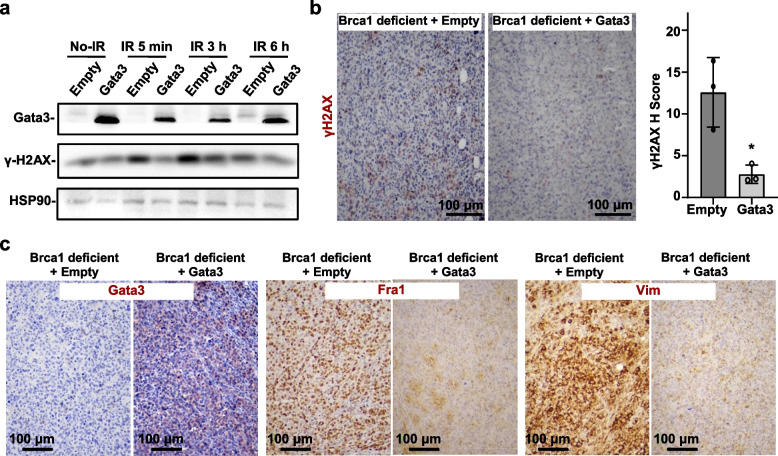
Reconstitution of GATA3 in BRCA1-deficient tumor cells reduces DNA damage. **a** *p18*^*−/−*^*;Brca1*^*MGKO*^ mammary tumor cells infected with pLVX-Flag (Empty) or pLVX-Flag-Gata3 (Gata3) were treated with or without ionizing radiation (IR) of 5 Gy, and the expression of Gata3 and γ-H2AX were analyzed at different time points after IR treatment. **b**, **c** *p18*^*−/−*^*;Brca1*^*MGKO*^ mammary tumor cells infected with infected with pBabe-puro-Gata3 (Gata3) and pBabe-puro-empty (Empty) were transplanted into left and right inguinal MFP of mice. Four weeks later, the regenerated mammary tumors were analyzed by IHC with antibodies against γH2AX (**b**) Gata3, Fra1, or Vim (**c**). The *H*-scores for γH2AX in **b** were calculated. The results represent the mean ± SD of three individual tumors per group. The asterisk (*) denotes a statistical significance from empty- and Gata3-overexpressed samples determined by the *T*-test. The blots and images represent data from at least three independent experiments or tumors

Prompted by the data that the loss of function of GATA3 generates similar phenotypes with that of BRCA1 with respect to impairing DNA damage repair (Figs. 1, 2, 3, and 4) and that GATA3 functions downstream of BRCA1 to suppress aberrant mammary cell differentiation [31, 32], we then examine whether GATA3 also functions downstream of BRCA1 to control DNA repair. We took advantage of mammary tumor cell lines derived from *p18*^*−/−*^*;Brca1*^*MGKO*^ mice, in which both Brca1 and Gata3 were undetectable [12, 32]. We transduced *p18*^*−/−*^*;Brca1*^*MGKO*^ mammary tumor cells with pLVX-Flag or pLVX-Flag-Gata3 or transfected human MDA-MB231 cells with pBabe-Empty or pBabe-GATA3 and then treated them with ionizing radiation (IR) or VP16. We found that after IR or VP16 treatment, the level of γ-H2AX in Gata3-overexpressing cells was significantly lower than the level of γ-H2AX in control cells (Fig. 5a, Additional file 1: Fig. S6a, S6c). Although overexpression of Gata3 induced the expression of CtIP, it is noteworthy that IR or VP16 treatment did not clearly alter its expression in Gata3-overexpressing cells (Additional file 1: Fig. S6b, S6c). In addition, we also confirmed that overexpression of Gata3 did not change the expression of RNF-8 and RAD51 in response to IR or VP16 (Additional file 1: Fig. S6b, 6c). These results suggest that Gata3 protects Brca1-deficient tumor cells from IR-induced DNA damage. We generated Gata3-overexpressing *p18*^*−/−*^*;Brca1*^*MGKO*^ mammary tumor cells and transplanted them into the MFPs of mice. We found that tumors generated by Gata3-overexpressing cells were smaller in size, as previously reported [32], and displayed significantly less γ-H2AX-positive cells than tumors generated by control cells (Fig. 5b, c, Additional file 1: Fig. S7). Notably, Gata3-overexpressing tumors expressed drastically less mesenchymal markers, Vim and Fra1, than control tumors (Fig. 5c). Taken together, these data indicate that reconstitution of Gata3 in *Brca1*-deficient cancer cells restores the efficiency of DNA damage repair and suppresses mesenchymal differentiation in inhibition of tumorigenesis.

### GATA3 promotes HR-mediated DSB repair and restores HR efficiency of Brca1-deficient cells

**Fig. 6:**
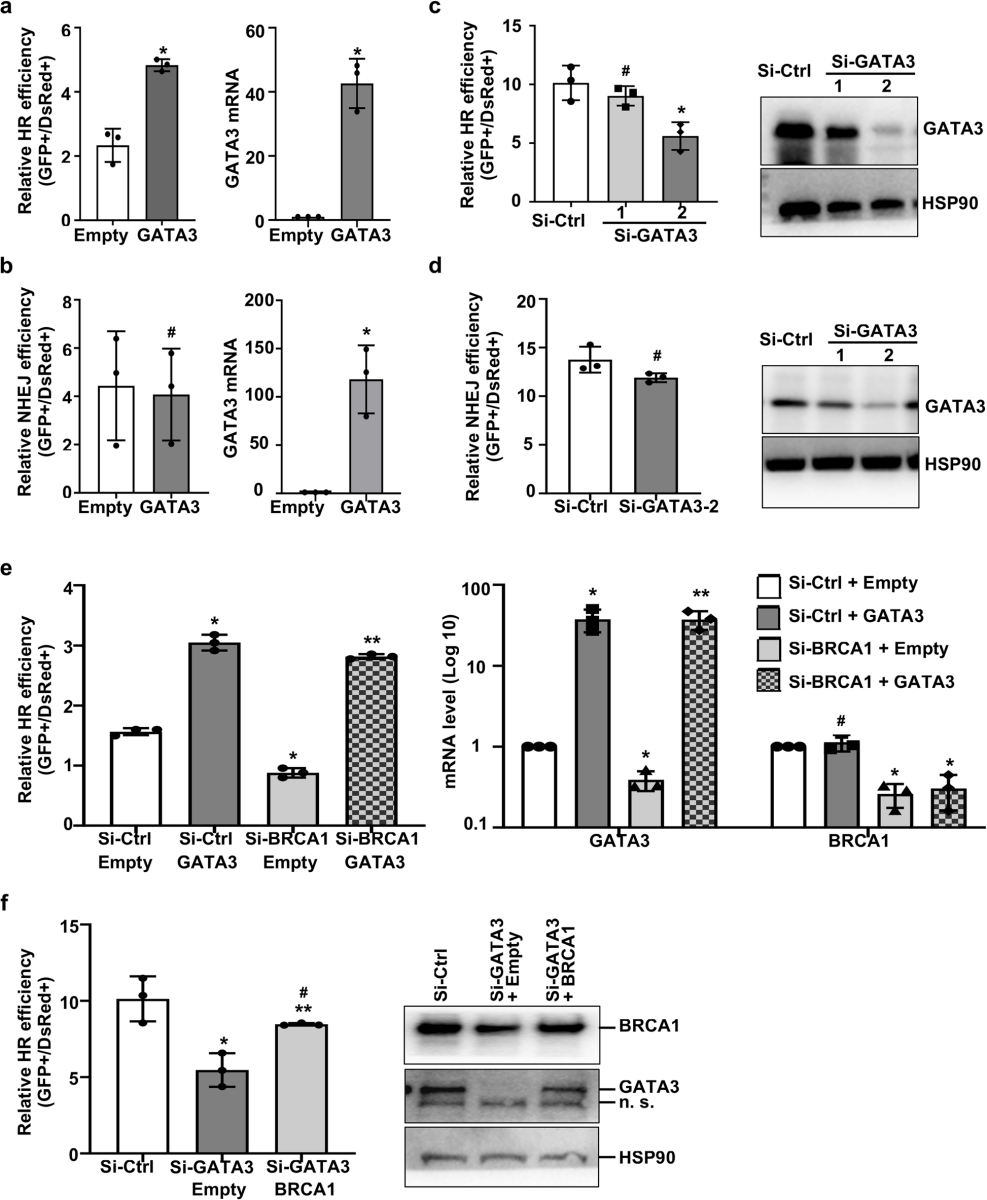
GATA3 promotes HR-mediated DNA damage repair and restores the efficiency of HR in Brca1-deficient cells. **a**, **b** The HR and NHEJ reporter cell lines, DR-GFP U2OS (**a**) and EJ5-GFP U2OS (**b**), were transfected with pBabe-Empty (Empty) or pBabe-GATA3 (GATA3), and then infected with virus expressing I-SceI. Two days later, cells were collected and analyzed for GFP expression by FACS (left panels) or for expression of GATA3 by qRT-PCR (right panels). **c**, **d** DR-GFP U2OS (**c**) or EJ5-GFP U2OS (**d**) cells were transfected with si-GATA3-1, si-GATA3-2 or si-control (si-Ctrl), infected with I-SceI, and analyzed for GFP (left) and GATA3 (right). The results in **a**–**d** represent the mean ± SD of three independent experiments. The asterisk (*) denotes a statistical significance from Empty and GATA3 or si-Ctrl and Si-GATA3 samples determined by the *T*-test. The number sign (#) denotes a statistical insignificance from Empty and GATA3 or si-Ctrl and Si-GATA3 samples determined by the *T*-test. **e** DR-GFP U2OS cells were firstly transfected with si-Ctrl or si-BRCA1 for 8 h and then transfected with pBabe-Empty (Empty) or pBabe-GATA3 (GATA3) for an additional 8 h. HR assay was done as above described (left) and gene expression was determined by qRT-PCR (right). **f** DR-GFP U2OS cells were firstly transfected with si-Ctrl or si-GATA3 for 8 h and then transfected with pBabe-Empty (Empty) or pBabe-BRCA1 (BRCA1) for an additional 8 h. HR assay was done as above described (left) and gene expression was determined by qRT-PCR (right). The results in **e** represent the mean ± SD of three independent experiments. The asterisk (*) denotes a statistical significance from Si-Ctrl + Empty and Si-Ctrl + GATA3, Si-BRCA1 + Empty, or Si-BRCA1 + GATA3 samples determined by the *T*-test. The asterisk (**) denotes a statistical significance from Si-BRCA1 + Empty and Si-BRCA1 + GATA3 samples determined by the *T*-test. The number sign (^#^) denotes a statistical insignificance from Si-Ctrl + Empty and Si-Ctrl + GATA3 samples determined by the *T*-test. The results in **f** represent the mean ± SD of three independent experiments. The asterisk (*) denotes a statistical significance from Si-Ctrl and Si-GATA3 + Empty samples determined by the *T*-test. The asterisk (**) denotes a statistical significance from Si-GATA3 + Empty and Si-GATA3 + BRCA1 samples determined by the *T*-test. The number sign (^#^) denotes a statistical insignificance from Si-Ctrl and Si-GATA3 + BRCA1 samples determined by the *T*-test

DNA DSBs are repaired by two major pathways: HR and NHEJ. To directly test which pathway GATA3 is involved in DNA damage repair, we carried out assays for HR- or NHEJ-mediated repair using the DR-GFP/I-Sce I or EJ5-GFP/I-Sce I system [40]. We transfected pBabe-Gata3 (Gata3) or pBabe-Empty (Empty) in HR and NHEJ reporter cell lines, which were then infected with a virus expressing I-SceI. We found that overexpression of GATA3 significantly stimulated HR by more than twofolds when compared with the HR of control cells (Fig. 6a). However, overexpression of GATA3 had no significant effect on NHEJ (Fig. 6b). In line with these results, we found that knockdown of GATA3 significantly reduced HR, but not NHEJ (Fig. 6c, d), consolidating that GATA3 promotes DNA damage repair by HR, but not NHEJ. We then focused on the role of GATA3 in BRCA1-mediated HR. We found that KD of BRCA1 significantly reduced the level of HR and the expression of GATA3 (Fig. 6e), confirming not only the role of BRCA1 in promoting HR, but also our previous finding that BRCA1 regulates GATA3 in mammary cells [32]. Notably, we observed that the HR efficiencies in si-BRCA1 + GATA3 and si-Control + GATA3 cells were comparable, both of which were significantly higher than the HR efficiencies in si-BRCA1 + Empty cells (Fig. 6e, left). The results indicate that reconstitution of GATA3 restores the HR efficiency of BRCA1-deficient cells. Next, we checked the role of BRCA1 in GATA3-mediated HR. We found that the HR efficiency in si-GATA3 + BRCA1 cells was significantly higher than that in si-GATA3 + Empty cells, but slightly lower than that in si-control cells (Fig. 6f, left), suggesting overexpression of BRCA1 restores HR efficiency of GATA3-deficient cells. Interestingly, we detected that the expression of GATA3 in si-GATA3 + BRCA1 cells was drastically enhanced relative to that in si-GATA3 + Empty cells (Fig. 6f, right), indicating that overexpression of BRCA1 induced GATA3 re-expression in GATA3 knockdown cells, which is likely responsible for the restoration of HR efficiency of GATA3-deficient cells. These results support that GATA3 functions downstream of BRCA1 to promote HR.

### Depletion of Gata3 sensitizes tumor cells to PARP inhibitor, and reconstitution of Gata3 promotes resistance of Brca1-deficient tumor cells to PARP inhibitor

**Fig. 7:**
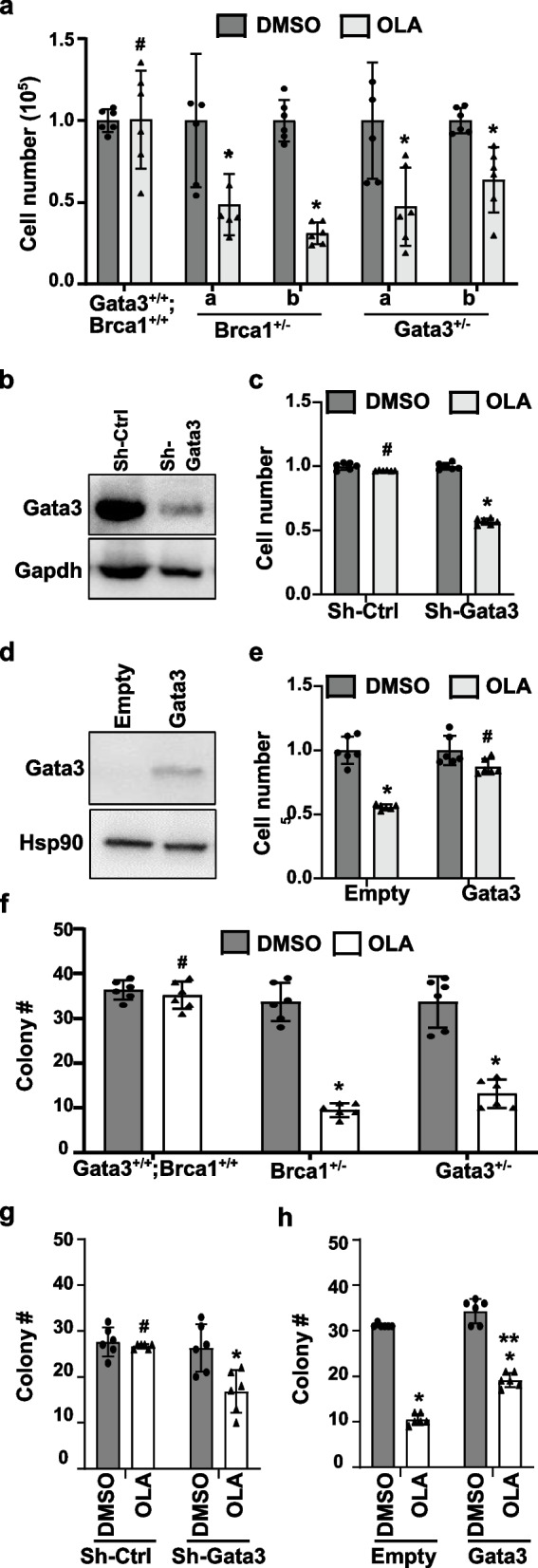
Gata3-deficient tumor cells are sensitive to PARPi and reconstitution of Gata3 de-sensitizes Brca1-deficient cells to PARPi. **a** MMTV-PyMT (*Gata3*^+/+^*;Brca1*^+/+^), *p18*^*−/−*^*;Gata3*^+*/−*^* (Gata3*^+*/−*^), and *p18*^*−/−*^*;Brca1*^+*/−*^* (Brca1*^+*/−*^) mammary tumor cells were treated with DMSO or OLA at 10 μM. Forty-eight hours later, the cell number was counted. **b**, **c** *Gata3*^+/+^*;Brca1*^+/+^ mammary tumor cells infected with psi-LVRU6GP-control (sh-Ctrl) or psi-LVRU6GP-Gata3 (sh-Gata3), selected with puromycin, and analyzed for Gata3 expression (**b**). *Gata3*^+/+^*;Brca1*^+/+^-sh-Ctrl and *Gata3*^+/+^*;Brca1*^+/+^-sh-Gata3 cells in **b** were treated with DMSO or OLA for 48 h; the cell number was counted (**c**). **d**, **e** *p18*^*−/−*^*;Brca1*^+*/−*^* (Brca1*^+*/−*^) mammary tumor cells infected with pLVX-Flag (Empty) or pLVX-Flag-Gata3 (Gata3), selected with hygromycin, and analyzed for Gata3 expression (**d**). Empty- and Gata3-expressing *Brca1*^+*/−*^ cells in (**d**) were treated with DMSO or OLA for 48 h; the cell number was counted (**e**). Data in **a**, **c**, and **e** represent the mean ± SD from duplicates of three independent experiments. The asterisk (*) denotes a statistical significance from DMSO- and OLA-treated samples determined by the *T*-test. The number sign (#) denotes a statistical insignificance from DMSO- and OLA-treated samples determined by the *T*-test. **f**–**h** MMTV-PyMT (*Gata3*^+/+^*;Brca1*^+/+^), *p18*^*−/−*^*;Gata3*^+*/−*^* (Gata3*^+*/−*^), and *p18*^*−/−*^*;Brca1*^+*/−*^* (Brca1*^+*/−*^) tumor cells (**f**), *Gata3*^+/+^*;Brca1*^+/+^ tumor cells infected with sh-Ctrl or sh-Gata3 (**g**), or *p18*^*−/−*^*;Brca1*^+*/−*^* (Brca1*^+*/−*^) tumor cells infected with pLVX-Flag (Empty) or pLVX-Flag-Gata3 (Gata3) (**h**) were treated with DMSO or OLA (1 μM) and analyzed by clonogenic assay. Data represent the mean ± SD from triplicates of two independent experiments. The asterisk (*) denotes a statistical significance from DMSO- and OLA-treated samples, and the asterisk (**) denotes a statistical significance from Flag and Gata3 samples treated with OLA. The number sign (#) denotes a statistical insignificance from DMSO- and OLA-treated samples determined by the *T*-test

We have previously demonstrated that GATA3 is regulated by BRCA1 and GATA3 functions downstream of BRCA1 to control mammary cell differentiation [31, 32]. The findings that GATA3 stimulates DSB repair through HR and reconstitution of Gata3 restores the HR efficiency of *Brca1*-deficient cancer cells prompting us to investigate the response of Gata3-deficient tumor cells to PARP inhibitor. We treated *Brca1*^+/−^ or *Gata*3^+/−^ mammary tumor cells with olaparib (OLA), a PARP inhibitor. We noticed that the number of *Brca1*^+/−^ or *Gata*3^+/−^ tumor cells, but not the number of *Gata3*^+/+^*;Brca1*^+/+^ tumor cells, was significantly reduced after OLA treatment (Fig. 7a). In addition, OLA treatment of Gata3 knockdown tumor cells, not control cells, also resulted in a significant reduction of the cell number (Fig. 7b, c). These data support that depletion of Gata3, like that of Brca1, in mammary tumor cells impairs HR-mediated DNA DSB repair.

We ectopically overexpressed Gata3 in *Brca1*-deficient mammary tumor cells and then treat them with OLA. In contrast to the significant decrease in the number of Flag-expressing Brca1-deficient tumor cells, the number of Gata3-expressing Brca1-deficient tumor cells was insignificantly changed in response to OLA (Fig. 7d, e). This result indicates that reconstitution of Gata3 rescues defects of HR-mediated DNA DSBs repair in Brca1-deficient cells, which is responsible for the resistance of the cells to PARP inhibitor.

We then performed cologenic assays to further evaluate the sensitivity of the cells to PARPi and found that OLA slightly reduced the colony formation of *Brca1*^+/+^;Gata3^+/+^ tumor cells but significantly inhibited the colony formation of *Brca1*^+/−^ or *Gata3*^+/−^ tumor cells (Fig. 7f, Additional file 1: Fig. S8). OLA treatment of sh-Gata3-*Brca1*^+/+^*;Gata3*^+/+^ tumor cells, not sh-Ctrl-*Brca1*^+/+^*;Gata3*^+/+^ tumor cells, again significantly reduced the number of colonies formed (Fig. 7g, Additional file 1: Fig. S8). Though OLA treatment of either Flag- or Gata3-expressing Brca1-deficient tumor cells resulted in significantly less colonies than DMSO treatment, the colonies formed by Gata3-expressing cells were significantly more than those formed by Flag-expressing cells (Fig. 7h, Additional file 1: Fig. S8). These results are consistent with the data derived from the cell proliferation assay and suggest that overexpression of GATA3 partially restores PARPi resistance in BRCA1-deficient cells.

## Discussion

In this study, we discovered that depletion of Gata3, like that of Brca1, impairs DNA damage repair in breast cancer cells in vitro and in vivo. Reconstitution of Gata3 improves DNA damage repair in Brca1-deficient mammary tumorigenesis. Overexpression of GATA3 promotes HR-mediated DSB repair and restores the HR efficiency of BRCA1-deficient cells. We further demonstrated that depletion of Gata3 sensitizes tumor cells to PARP inhibitor, and reconstitution of Gata3 enhances resistance of Brca1-deficient tumor cells to PARP inhibitor. Together with our previous findings, these results demonstrate that GATA3 functions downstream of BRCA1 controlling HR-mediated DNA damage repair and aberrant differentiation. Our findings suggest that PARP inhibitors are effective for the treatment of GATA3-deficient BLBCs.

Unrepaired or improperly repaired DNA damage is responsible for genome instability that is associated with a predisposition to various types of cancer including breast cancer and grades of cancer cell differentiation [41]. Inadequate DNA damage repair resulting from the depletion of BRCA1 or its interacting proteins including FANCD2, BRG1, NUMB, and HES1 promotes aberrant luminal to basal and mesenchymal differentiation in immortalized mammary epithelial cells. However, depletion of BRCA2, another BRCA1-interacting protein, impairs DNA damage repair [3, 4], but does not cause aberrant differentiation, suggesting that not all BRCA1-interacting proteins that functions in DNA damage repair regulate mammary cell differentiation. These discoveries prompt us to determine if transcription factors that control mammary cell differentiation play a role in DNA damage repair.

GATA3, a BRCA1-interacting transcription factor, is required for differentiation of mammary luminal epithelial cells [6, 19]. GATA3 is highly expressed in luminal breast cancers [23, 24], but hardly detectable in BLBCs [6, 25]. We previously reported that GATA3 functions downstream of BRCA1 to inhibit luminal to basal and mesenchymal trans-differentiation in suppression of BLBC development and progression [31, 32]. Not until recently has the function of GATA3 loss in regulating DNA damage repair been noticed. Depletion of GATA3 in MCF7 breast cancer cells reduces the expression of CtIP, an essential protein involved in HR, and impairs DNA damage response [33]. Overexpression of WT GATA3, but not a few mutant GATA3 proteins including GATA3 D335E mutant, transactivates CtIP and promotes HR [33]. Since MCF7 cells harbor a heterozygous GATA3 D335E mutant allele, they express barely detectable WT GATA3 and a large amount of mutant GATA3 [22, 32, 36]. The finding that knockdown of GATA3 in MCF7 cells reduces CtIP expression and impairs DNA damage response [33] does not exclude the possibility that the reduced CtIP and DNA damage response resulted from the depletion of mutant, not WT, GATA3 in these cells. Therefore, the finding observed in MCF7 cells that the loss of function of GATA3 impairs DNA damage repair remains to be confirmed. In the present study, we discovered that spontaneously developed mammary tumors from *p18*^−/−^*;Gata3*^+/−^ mice displayed a significantly increased number of cells with DNA DSBs when compared with the tumors from *p18*^−/−^ mice. Knockdown of Gata3 in MMTV-PyMT luminal tumor cells also drastically enhanced the number of cells with DNA DSBs in tumorigenesis. Knockdown of GATA3 in human T47D (GATA3 WT) breast cancer cells or haploid loss of Gata3 by heterozygous germline deletion of Gata3 gene in mouse mammary tumor cells impaired DNA damage response in vitro. Haploid loss of Gata3 promoted estrogen-induced DNA damage in mammary tumorigenesis. Notably, as controls, in most of the above experimental systems, we noticed similar defects in DNA damage repair in Brca1-deficient tumor cells, though the grade of the defects in DNA damage repair in Gata3-deficient cells was not as strong as the grade of the defects in Brca1-deficient cells. In addition, we found that knockdown of GATA3 in T47D cells downregulates CtIP expression, confirming that GATA3 positively regulates the expression of CtIP. Together, our data demonstrate that depletion of GATA3 impairs DNA damage response in mammary tumor cells in vitro and in mammary tumorigenesis in vivo.

Interaction among BRCA1, GATA3, and CtIP in DNA damage repair and mammary cell differentiation is quite interesting. GATA3 stimulates the expression of CtIP and GATA3 itself is regulated by BRCA1 [32, 33]. Both GATA3 and CtIP are BRCA1-interacting proteins [1, 19, 42]. BRCA1-CtIP complex plays a critical/central role in HR-mediated DNA DSB repair [42], while BRCA1-GATA3 complex protects mammary cells from aberrant differentiation [12, 19, 32]. Interestingly, heterozygous germline deletion and mammary epithelial cell-specific deletion of CtIP do not result in the spontaneous development of mammary tumors, and loss of CtIP function may inhibit mammary tumor formation in p53-deficient mice [43]. However, since the gene targeting of CtIP is not confirmed [43] and the mammary tumors developed in p53-deficient mice are typical BLBCs with EMT features [44], it remains elusive whether CtIP plays a role in regulating luminal to basal or mesenchymal differentiation in normal or cancerous mammary cells. We previously discovered that Brca1 positively regulates GATA3 expression and that GATA3 functions downstream of BRCA1 suppressing luminal-to-basal and to mesenchymal differentiation in mammary cells as well as tumor development and progression [31, 32]. We found in this study that overexpression of GATA3 significantly promotes HR. Reconstitution of Gata3 in Brca1-deficient mammary tumor cells restores the HR efficiency of Brca1-deficient cells and improves DNA damage repair in mammary tumorigenesis. Depletion of Gata3, similar to Brca1 depletion, sensitizes tumor cells to PARP inhibitor-induced cell death. Reconstitution of Gata3 enhances resistance of Brca1-deficient tumor cells to PARP inhibitor. Taking into consideration our previous discovery that Brca1 positively regulates GATA3 expression and GATA3 functions downstream of BRCA1 suppressing luminal-to-basal and mesenchymal differentiation in mammary cells as well as tumor development and progression [31, 32], our findings demonstrate that GATA3 functions downstream of BRCA1 to promote HR-mediated DNA damage repair and to suppress aberrant differentiation in mammary and tumor development.

## Conclusions

These results demonstrate that Gata3 functions downstream of BRCA1 to promote DNA damage repair and suppress dedifferentiation in mammary tumorigenesis and progression. Our findings suggest that PARP inhibitors are effective for the treatment of GATA3-deficient BLBCs.

## Methods

### Mice, histopathology, immunostaining, and loss of heterozygosity (LOH) analysis

The generation of *p18*^−*/*−^*, p18*^−*/*−^*;Gata3*^+*/*−^*, p18*^−*/*−^*;Brca1*^+*/*−^*, and p18*^−*/*−^*;Brca1*^*MGKO*^* (p18*^−*/*−^*;Brca1*^*f/f*^*;MMTV-Cre* or *p18*^−*/*−^*;Brca1*^*f/*−^*;MMTV-Cre)* mice was previously described [12, 25, 35, 45]. Female NSG mice were purchased from Jackson Laboratory (Bar Harbor, ME). Female NCG and FVB/NJGpt-Tg (MMTV-PyMT)/Gpt were purchased from GemPharmatech (Nanjing, China). The Institutional Animal Care and Use Committee at the University of Miami and Shenzhen University approved all animal procedures. Animals were housed in a specific pathogen-free environment. The investigators were not blinded to genotype allocation during experiments and outcome assessment. No randomization method was used as mice were segregated into groups based on genotype. Histopathology and immunohistochemistry (IHC) were performed as previously described [12, 25, 35]. The primary antibodies used were as follows: γH2AX, GATA3 (Cell Signaling), Ck5 (Covance), and Ck8 (American Research Products). Immunocomplexes were detected using the Vectastain ABC alkaline phosphatase kit according to the manufacturer’s instructions (Vector Laboratories) or using FITC- or rhodamine-conjugated secondary antibodies (Jackson Immunoresearch). The positive results of γH2AX staining were quantified using H-score as previously described [39, 46]. Briefly, scores are generated by adding together 3 × % strongly stained nuclei, 2 × % moderately stained nuclei, and 1 × % weakly stained nuclei, giving a possible range of 0 to 300. LOH analysis was performed as we previously described [35]. Briefly, 10-µm sections were deparaffinized and lightly stained with hematoxylin. The tumors that were clearly separated from normal tissues were isolated from the slides. DNA was isolated from the tissue samples and analyzed.

### Mammary tumor cell preparation, transplantation, and tumor treatment

Mammary tumors were dissected from female mice and tumor cell suspensions were prepared as previously described [12, 25, 35]. For mammary tumor cell transplantation, cells were suspended in a 50% solution of Matrigel (BD) and then inoculated into the left and/or right inguinal mammary fat pads (MFPs) of 4–6-week-old NSG or NCG mice, respectively. Six or 7 weeks after transplantation, animals were euthanized and mammary tumors were dissected for histopathological, immunohistochemical, and biochemical analyses. For estrogen treatment in vivo, 0.72 mg E2 (SE-121, IRA, Sarasota, FL, USA) or Beeswax pellet was implanted subcutaneously in mice receiving tumor cell or tissue transplants.

### Cell culture, treatment, clonogenic assay, overexpression and knockdown of GATA3, and knockdown of BRCA1

T47D cells purchased from ATCC were tested and authenticated as previously described [12]. T47D cells were cultured per ATCC recommendations. Primary murine mammary tumor cells were cultured in 10% FBS (Gibco). For drug treatment, cells were cultured in the presence of Olaparib (OLA, Sigma), etoposide (VP16, Sigma), 17β-estradiol (E2, Sigma), or DMSO for different times and then were lysed for further analysis, or counted for the cell number by an automatic cell counter (Olympus). For stable overexpression of Gata3 in mouse cells, cells were infected with pLVX-3Flag (Flag) and pLVX-3Flag-Gata3 (Gata3), respectively, and selected with hygromycin, as previously described [32]. For transient overexpression of GATA3 or BRCA1 in human cells, cells were transfected with pBabe-GATA3 (GATA3), pBabe-BRCA1 (BRCA1), or pBabe-empty (Empty), as previously described [32]. For stable knockdown (KD) of GATA3 in human cells, T47D cells were infected with pGIPZ-empty, pGIPZ-shGATA3-E9 (shGATA3-1), and pGIPZ-shGATA3-A12(shGATA3-2), as previously described [12]. For stable knockdown of Gata3 in murine cells, MMTV-PYMT tumor cells were infected with psi-LVRU6GP-control, psi-LVRU6GP-Gata3-a, or psi-LVRU6GP-Gata3-c (GeneCopoeia, Guangzhou, China), then selected with puromycin, as previously described [31]. For transient knockdown of BRCA1 or GATA3 in human cells, the following siRNA oligonucleotide duplexes were used as we described [30, 47]: BRCA1 (5′-CUAGAAAUCUGUUGCUAUGdTdT-3′), GATA3 (siRNA-1: 5′-TGCCTGTGGGCCTTACTACdTdT-3′, siRNA-2: 5′-CATCGACGGTCAAGGCAACdTdT-3′, siRNA-3: 5′-GGGCUCUACUACAAGCUUCdTdT-3′), Control (5′-UUCUCCGAACGUGUCACGUdTdT-3′). Clonogenic assay was performed as previously described [48]. Briefly, 200 tumor cells were seeded into six-well plates, treated with DMSO or OLA for 10 days, and then fixed with 4% paraformaldehyde. The colonies were stained with 0.2% crystal violet for 30 min. The number of colonies consisting of 50 or more cells was counted.

### Western blot and qRT-PCR

Western blot analysis was carried out as previously described [12, 31, 32]. Primary antibodies used are as follows: HSP90, Gapdh (Ambion), γH2AX, GATA3, CtIP (Cell Signaling). For qRT-PCR, total RNA was extracted using TRIzol (Invitrogen) according to the manufacturer’s protocol and cDNA was generated using the RT Kit (YEASEN, Shanghai, China). qRT-PCR was performed as previously reported [35].

### HR and NHEJ repair assays

The green fluorescent protein (GFP) reporter system for HR-mediated DSB repair (DR-GFP U2OS cells), the GFP reporter system for NHEJ-mediated DSB repair (EJ5-GFP U2OS cells), and the I-SceI expression construct were generous gifts from Maria Jasin (Memorial Sloan Kettering Cancer Center). HR assay was performed as described [40]. Briefly, DR-GFP U2OS cells were transfected with pBabe-GATA3, pBabe-BRCA1, pBabe-Empty, or siRNA oligonucleotide duplexes for 8 h, and 8 h later infected with virus expressing I-SceI. DR-GFP U2OS cells were transfected with siRNA oligonucleotide duplexes for 8 h, then transfected with pBabe-GATA3, pBabe-BRCA1, or pBabe-Empty for 8 h, and lastly, infected with virus expressing I-SceI. Two days after infection by I-SceI, the fraction of GFP-positive cells was determined by FACS. NHEJ assay was done as described [49]. Briefly, EJ5-GFP U2OS cells were transfected with pBabe-GATA3, pBabe-Empty, or siRNA oligonucleotide duplexes for 8 h. Eight hours later, the cells were infected with I-SceI. Two days later, GFP-positive cells were determined by FACS.

### Statistical analysis

All data are presented as the mean ± SD for at least three repeated individual experiments for each group. Sample sizes, number of replicates, and normalization methods are indicated in each figure legend. No statistical methods were used to pre-determine sample/group sizes. Statistical analyses were performed as described in each figure legend using GraphPad PRISM 6.02 software. Quantitative results were analyzed by two-tailed Student’s *t* test. *p* < 0.05 was considered statistically significant.

### Supplementary Information

**Additional file 1:** **Fig. S1. **Analysis of cell proliferation in primary mammary tumors. Representative mammary tumors were analyzed by IHC with an antibody against Ki-67. Two independent mammary tumors derived from *p18*^-/-^, *p18*^-/-^;*Gata3*^+/-^ and *p1*8^-/-^*;Brca1*^+/-^ mice individually are shown. **Fig. S2.** Depletion of Gata3 in luminal tumor cells enhances DNA damage in tumorigenesis. MMTV-PyMT luminal tumor cells infected with psi-LVRU6GP-control (sh-Ctrl) or psi-LVRU6GP-Gata3-c (sh-Gata3) were transplanted into the left and right inguinal mammary fat pads of female mice. Tumors formed 8 weeks after transplantation were analyzed by IHC with an antibody against γ-H2AX. Representative analysis of two pairs of tumors is shown.** Fig. S3. **Analysis of GATA3 deficient T47D breast cancer cells treated with VP16. (a) T47D-Si-Ctrl, T47D-Si-GATA3-1, and T47D-Si-GATA3-2 cells were treated with VP16 at a concentration of 10 μM for 0.5 hours, allowed to recover for 0 or 20 hours, and then immunostained with antibodies against γH2AX and cyclin A. (b) Quantification of Cyclin A positive cells in (a). At least 200 cells were counted for each sample. The results represent the mean ± SD of five randomly selected fields per group. (c) Quantification of protein levels of γH2AX and CtIP in Fig. 3a.** Fig. S4. **Depletion of GATA3 in breast cancer cells impairs DNA damage repair *in vitro*. T47D-Si-Ctrl, T47D-Si-GATA3-1, and T47D-Si-GATA3-2 cells were treated with VP16 at a concentration of 10 μM for 0.5 hours, allowed to recover for 0 or 20 hours, and then immunostained with an antibody against 53BP1. Note relative to T47D-Si-Ctrl cells, T47D-Si-GATA3-1 and T47D-Si-GATA3-2 cells retain high level of 53BP1 after 20-hour recovery.** Fig. S5. **Depletion of GATA3 in U2OS cells impairs DNA damage repair* in vitro*. (a) U2OS cells were transfected with Si-GATA3-1, Si-GATA3-2, or Si-control (Si-Ctrl) and analyzed. (b) U2OS-Si-Ctrl, U2OS-Si-GATA3-1, and U2OS-Si-GATA3-2 cells were treated with VP16 at a concentration of 10 μM for 0.5 hours, allowed to recover for 0 or 20 hours, and then immunostained with antibodies against γH2AX and 53BP1. Representative γH2AX- and 53BP1-positive cells were shown. (c) Quantification of γH2AX positive cells in (b). Only cells with at least 3 γH2AX foci were counted as positive cells. At least 200 cells were counted for each sample. (d) Quantification of 53BP1 positive cells. The results in (c) and (d) represent the mean ± SD of five randomly selected fields per group. The asterisk (*) denotes a statistical significance from Si-Ctrl and Si-GATA3-1 or Si-GATA3-2 samples determined by the T-test.** Fig. S6.** Overexpression of GATA3 reduces the expression of γ-H2AX and enhances the expression of CtIP in mammary tumor cells. (a) γ-H2AX levels of each lane in Fig. 5a were quantified, respectively, by Image-Pro Plus 6.0 and normalized by that of HSP90. (b) *p18*^-/-^*;Brca1*^MGKO^ mouse mammary tumor cells infected with pLVX-Flag (Empty) or pLVX-Flag-Gata3 (Gata3) were treated with or without ionizing radiation (IR) of 5 Gy, and the expression of the genes indicated were analyzed by Western blot. (c) Human MDA-MB231 breast cancer cells transfected with pBabe-Empty (Empty) or pBabe-GATA3 (GATA3) were treated with or without VP16 for 0.5 hours, and the expression of the genes indicated were analyzed by Western blot (left). γ-H2AX levels of each lane in (c, left) were quantified, respectively, by Image-Pro Plus 6.0 and normalized by that of GAPDH (right).** Fig. S7. **Reconstitution of GATA3 restores DNA repair in BRCA1 deficient tumor cells. *p18*^-/-^*;Brca1*^MGKO^ mammary tumor cells infected with pBabe-puro-Gata3 (Gata3) and pBabe-puro-empty (Empty) were transplanted into  left and right inguinal MFP of mice. Four weeks later, the regenerated mammary tumors were analyzed by IHC.** Fig. S8. **Analysis of the response of mammary tumor cells to OLA. (a). 200 *Brca1*^+/+^*;Gata3*^+/+^, *Brca1*^+/-^ or* Gata3*^+/-^ cells per well were seeded and treated with DMSO or OLA (1 μM). Ten days later, the colonies were fixed and stained with 0.2% crystal violet. (b, d) 200 Sh-Ctrl-*Brca1*^+/+^*;Gata3*^+/+^ and Sh-Gata3-*Brca1*^+/+^;*Gata3*^+/+^ (b) or Empty-*Gata3*^+/-^ and Gata3-*Gata3*^+/-^(d) cells per well were seeded and treated with DMSO or OLA (1 μM). Ten days later, the colonies were fixed and stained with 0.2% crystal violet. (c, e) Sh-Ctrl-*Brca1*^+/+^;*Gata3*^+/+^ and Sh-Gata3-*Brca1*^+/+^*;Gata3*^+/+^ (c) or Empty-*Gata3*^+/-^ and Gata3-*Gata3*^+/-^(e) cells were analyzed for the expression of GATA3. **Fig. S9.** Uncropped pictures of blots.

**Additional file 2.** Supporting data values. 

## Data Availability

All data generated or analyzed during this study are included in this published article and its supplementary information files (Additional files 1 and 2).

## Abbreviations

BLBCs: Basal-like breast cancers
EMT: Epithelial-mesenchymal transition
PARPi: PARP inhibitor
DSBs: Double-strand breaks
HR: Homologous recombination
NHEJ: Nonhomologous end joining
ER: Estrogen receptor
PARP: Poly (ADP-ribose) Polymerase
MECs: Mammary epithelial cells
MFPs: Mammary fat pads
OLA: Olaparib
VP16: Etoposide
E2: 17β-Estradiol
