# Supplementary material for: GATA3 functions downstream of BRCA1 to promote DNA damage repair and suppress dedifferentiation in breast cancer
Source: BMC Biol. 2024 Apr 16;22:85. doi: 10.1186/s12915-024-01881-6 (PMC11020915; doi:10.1186/s12915-024-01881-6)
Supplement: Supplementary file 1 — Additional file 1: Fig. S1. Analysis of cell proliferation in primary mammary tumors. Representative mammary tumors were analyzed by IHC with an antibody against Ki-67. Two independent mammary tumors derived from p18-/-, p18-/-;Gata3+/- and p18-/-;Brca1+/- mice individually are shown. Fig. S2. Depletion of Gata3 in luminal tumor cells enhances DNA damage in tumorigenesis. MMTV-PyMT luminal tumor cells infected with psi-LVRU6GP-control (sh-Ctrl) or psi-LVRU6GP-Gata3-c (sh-Gata3) were transplanted into the left and right inguinal mammary fat pads of female mice. Tumors formed 8 weeks after transplantation were analyzed by IHC with an antibody against γ-H2AX. Representative analysis of two pairs of tumors is shown. Fig. S3. Analysis of GATA3 deficient T47D breast cancer cells treated with VP16. (a) T47D-Si-Ctrl, T47D-Si-GATA3-1, and T47D-Si-GATA3-2 cells were treated with VP16 at a concentration of 10 μM for 0.5 hours, allowed to recover for 0 or 20 hours, and then immunostained with antibodies against γH2AX and cyclin A. (b) Quantification of Cyclin A positive cells in (a). At least 200 cells were counted for each sample. The results represent the mean ± SD of five randomly selected fields per group. (c) Quantification of protein levels of γH2AX and CtIP in Fig. 3a. Fig. S4. Depletion of GATA3 in breast cancer cells impairs DNA damage repair in vitro. T47D-Si-Ctrl, T47D-Si-GATA3-1, and T47D-Si-GATA3-2 cells were treated with VP16 at a concentration of 10 μM for 0.5 hours, allowed to recover for 0 or 20 hours, and then immunostained with an antibody against 53BP1. Note relative to T47D-Si-Ctrl cells, T47D-Si-GATA3-1 and T47D-Si-GATA3-2 cells retain high level of 53BP1 after 20-hour recovery. Fig. S5. Depletion of GATA3 in U2OS cells impairs DNA damage repair in vitro. (a) U2OS cells were transfected with Si-GATA3-1, Si-GATA3-2, or Si-control (Si-Ctrl) and analyzed. (b) U2OS-Si-Ctrl, U2OS-Si-GATA3-1, and U2OS-Si-GATA3-2 cells were treated with VP16 at a concentration of [file 12915_2024_1881_MOESM1_ESM.zip › Additional file 1 Fig. S8.pdf]

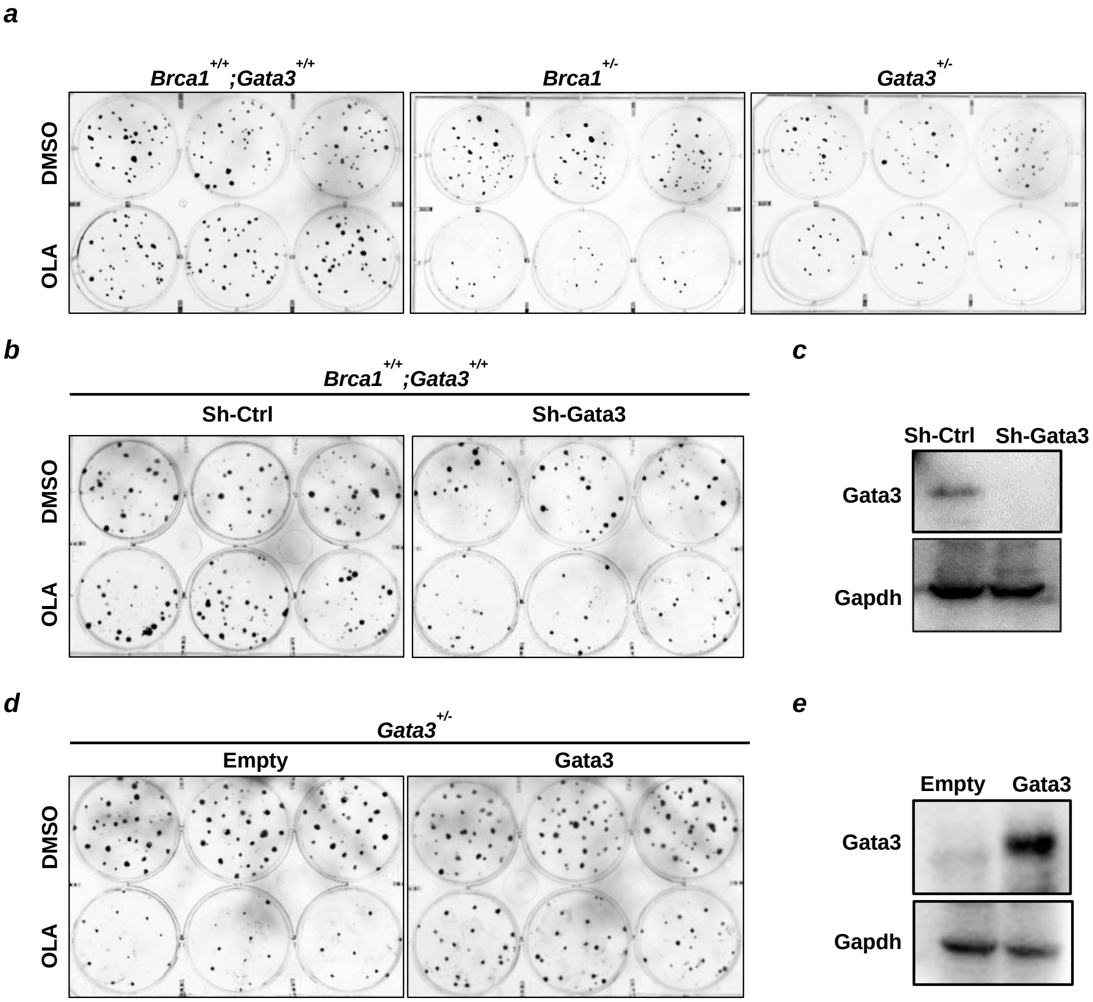

Fig. S8. Analysis of the response of mammary tumor cells to OLA. (a). 200 *Brca1*$^{+/+}$*;Gata3*$^{+/+}$, Brca1$^{+/-}$ or *Gata3*$^{+/-}$ cells per well were seeded and treated with DMSO or OLA (1 μM). Ten days later, the colonies were fixed and stained with 0.2% crystal violet. (b, d) 200 Sh-Ctrl-*Brca1*$^{+/+}$*;Gata3*$^{+/+}$ and Sh-Gata3-*Brca1*$^{+/+}$*;Gata3*$^{+/+}$ (b) or Empty-*Gata3*$^{+/-}$ and Gata3-*Gata3*$^{+/-}$(d) cells per well were seeded and treated with DMSO or OLA (1 μM). Ten days later, the colonies were fixed and stained with 0.2% crystal violet. (c, e) Sh-Ctrl-*Brca1*$^{+/+}$*;Gata3*$^{+/+}$ and Sh-Gata3-*Brca1*$^{+/+}$*;Gata3*$^{+/+}$ (c) or Empty-*Gata3*$^{+/-}$ and Gata3-*Gata3*$^{+/-}$(e) cells were analyzed for the expression of GATA3.
